# The relationship between menopausal syndrome and gut microbes

**DOI:** 10.1186/s12905-022-02029-w

**Published:** 2022-11-08

**Authors:** Yaqian Liu, Ying Zhou, Ting Mao, Yanmei Huang, Jingtao Liang, Min Zhu, Peixun Yao, Yun Zong, Jianying Lang, Yingxuan Zhang

**Affiliations:** 1 Gynecology, Guangzhou University of Traditional Chinese Medicine First Affiliated Hospital, Guangzhou, China; 2 The First Clinical College, Guangzhou University of Chinese Medicine, Guangzhou, China; 3 College of Basic Medicine, Guangzhou University of Chinese Medicine, Guangzhou, China; 4 Division of Laboratory Medicine, Zhujiang Hospital, Southern Medical University Guangzhou, Guangzhou, China

**Keywords:** Menopausal syndrome, Gut microbes, 16S ribosomal RNA gene sequencing, Gut microbiota dysbiosis, Functional prediction

## Abstract

**Background:**

Gut microbes were closely related to women’s health. Previous studies reported that the gut microbes of premenopausal women were different from those of postmenopausal women. However, little was known about the relationship between gut microbiota dysbiosis and menopausal syndrome (MPS). The aim of this study was to explore the relationship between MPS and gut microbes.

**Methods:**

Patients with MPS (P group, *n* = 77) and healthy women (H group, *n* = 24) at menopause were recruited in this study. The stool specimen and clinical parameters (demographic data, follicle stimulating hormone (FSH), luteinizing hormone (LH), estradiol (E2), et al) of participants’ were collected. We evaluated the differences in gut microbes by 16S ribosomal RNA gene sequencing. We used LEfSe to identify gut microbes with varying abundances in different groups. The Spearman correlation coefficients of clinical parameters and gut microbes were calculated. PICRUSt was used to predict the potential KEGG Ortholog functional profiles of microbial communities.

**Results:**

The abundance of 14 species differed substantially between the MPS and menopausal healthy women (LDA significance threshold > 2.0) according to LEfSe analysis. Using Spearman’s correlation analysis, it was discovered that E2 had a positive correlation with *Aggregatibacter segnis, Bifidobacterium animalis, Acinetobacter guillouiae* (*p* < 0.05, these three species were enriched in menopausal healthy women), while FSH and LH had a negative correlation with them (*p* < 0.05). KEGG level3 metabolic pathways relevant to cardiovascular disease and carbohydrate metabolism were enriched in the MPS (*p* < 0.05), according to functional prediction by PICRUST and analyzed by Dunn test.

**Conclusion:**

There was gut microbiota dysbiosis in MPS, which is reflected in the deficiency of the abundance of *Aggregatibacter segnis*, *Bifidobacterium animalis* and *Acinetobacter guillouiae* related to the level of sex hormones. In MPS individuals, species with altered abundances and unique functional pathways were found.

**Supplementary Information:**

The online version contains supplementary material available at 10.1186/s12905-022-02029-w.

## Background

During the menopause transition, women usually experience a progressive change in ovarian activity and a physiologic deterioration of hypothalamic-pituitary-ovarian axis function associated with fluctuating hormone levels [1], which cause menopausal syndrome (MPS). Hot flashes, night sweats, sleep disturbances, sexual dysfunction, mood disorders, and other symptoms are problematic symptoms experienced by women with MPS [2]. Menopausal symptoms affect 69.5% to 80% of women at menopause [3, 4]. Menopausal symptoms not only have a detrimental impact on one’s quality of life, but may also link patients to cardiovascular disease, diabetes, osteoporosis, and breast cancer [5–8]. As a result, the prevention and treatment of MPS demand more attention.

More and more studies showed that the gut microbes are closely related to health and disease. Gut microbes are regarded as one of the organs of human body. The intestine is colonized with 10^13^–10^14^ bacteria mainly residing in the lumen and in the mucus [9]. Gut dysbiosis is involved in many female reproductive and endocrine diseases, such as endometriosis [10], polycystic ovary syndrome [11], obesity and sexual precocious puberty [12], et al. Endometriosis is a chronic inflammatory disease, which is estrogen dependent. Gut microbes participate in the metabolism of estrogen in blood. The gut microbiota regulates estrogens through secretion of β-glucuronidase, an enzyme that deconjugates estrogens into their active forms [13]. The gut microbes in patients with endometriosis may have a large number of β- Glucuronidase producing bacteria, which may lead to increased levels of estrogen metabolites, leading to endometriosis [13]. Endometriosis is common in premenopausal women, and the disease may be mediated by high estrogen levels [14].

During menopause and postmenopause a variety of negative health outcomes may occur from the depletion of circulating estrogen. Premenopausal and postmenopausal women have different gut microbes [15]. A study showed that the gut microbiota community in menopausal women changed after isoflavones supplementation [16], which are plant-derived phytoestrogens. Another study showed that the level of estrogen metabolites in urine was positively correlated with the diversity of gut microbiota [17]. Osteoporosis develops as a result of continuous hypoestrogenemia. The gut microbiota has been found to interfere with hormone secretion, estrogen levels, metabolism and immune function, all of which influence bone metabolism [18, 19]. Numerous menopause-related symptoms and signs are derived as a result of lack of estrogen production. However, the link between gut microbiota and MPS was still poorly characterized. As a result, little was known regarding the link between gut dysbiosis and MPS. Furthermore, it remained unexplored how and which gut microbes may influence the key pathophysiological processes in MPS.

In order to study the taxonomic and functional features of the gut microbes in menopausal women, we conducted 16S ribosomal RNA (16S rRNA) gene sequencing and functional prediction analysis on the gut microbes of stool specimen taken from patients with MPS and healthy controls. Characterization of the compositional and functional features of the gut microbes in menopausal women helps us comprehend its involvement in women’s health, and hence the importance of gut microbes regulation in menopausal women’s health. We aim to provide potential techniques for the prevention and treatment of MPS.

## Methods

### Study participants

This study was a case-control study that included patients with menopausal syndrome (P group) and healthy women at menopause (H group). The Medical Ethics Committee of the Guangzhou University of Traditional Chinese Medicine First Affiliated Hospital approved this study (NO.ZYYECK【2020】021). From June 2020 to October 2021, we recruited females aged 40–60 to perform the domestic modified Kupperman index (KI) score (Additional file 1) test at our hospital. All participants were informed about the study’s purpose and given written informed consent.

Participants in the P group must satisfy the following criteria: (1) female, 40–60 years old; (2) have at least one of these autonomic nerve changes (hot flashes, night sweats, insomnia, irritability, and other symptoms) and menstrual disorders (two consecutive cycle length changes > 7 days in 10 months); (3) have a domestic modified KI score > 15 points.

Participants in the H group must meet the following requirements: (1) female, 40–60 years old; (2) with a domestic modified KI score < 15 points and no hot flashes.

The following were the exclusion criteria: (1) have unexplained irregular vaginal bleeding; (2) have used sex hormones within 3 months; (3) have antibiotics within 2 weeks; (4) have a history of severe, progressive, or uncontrolled cardiac, hepatic, renal, mental, or hematological diseases.

A total of 101 out of 1253 women who participated in the survey from June 2020 to October 2021 were included in the analysis to investigate the gut microbes of two groups after the exclusion of women who were aged under 40 years and over 60 years (*n* = 806), meet the exclusion criteria and refused toparticipate (*n* = 366). Lastly, we included 77 patients with menopausal syndrome (P group) and 24 healthy women at menopause (H group).

### Data collection

The Guangzhou University of Traditional Chinese Medicine First Affiliated Hospital used a survey (Additional file 1) and a physical examination to collect data on demographic characteristics, medical history, menstrual history, height, weight, body mass index (BMI), waist circumstance (WC), hip, waist hip ratio (WHR), and blood pressure. Participants were given a fecal collection kit and instructed to collect their feces within 1 week after their visit. Within 4 h of collection, feces samples were collected and sent to the hospital for examination. The samples were kept at − 80 °C until they were processed. After 8 hours of fasting, the H group had their blood obtained to check for follicle stimulating hormone (FSH), luteinizing hormone (LH), and estradiol (E2). After an 8-hour fast, the P group was required to have blood obtained and a bone mineral density (BMD) test. FSH, LH, E2, total cholesterol (CHOL), high-density lipoprotein cholesterol (HDL), low-density lipoprotein cholesterol (LDL), triglycerides (TG), fasting blood glucose (Glu), and fasting insulin (INS) are some of the blood test indications. The participants’ serum test results and BMD (dual-energy x-ray absorptiometry, DXA) testing room results were obtained from hospital laboratories and BMD (dual-energy x-ray absorptiometry, DXA) testing room, respectively.

### Diagnostic criteria of menopausal syndrome

Clinical manifestations, followed by sex hormone levels, are used to diagnose MPS. Diagnostic criteria referred to the Obstetrics and Gynecology Clinical Guidelines [20]. The following are the diagnostic criteria: (1) menstrual disorders are the first clinical symptoms of perimenopause; (2) vasomotor symptoms are primarily hot flashes; (3) may have one or more additional symptoms such as mental disorder (anxiety, depression, or insomnia), urogenital atrophy, cardiovascular symptoms (chest tightness or palpitations), skin and body changes (itchy skin, obesity), and osteoporosis; (4) FSH>10 IU/L indicates decreased ovarian reserve; amenorrhea, FSH>40 IU/L and E2<10-20pg/mL indicates ovarian failure.

### DNA extraction and PCR amplication

The HiPure Bacterial DNA Kit was used to extract bacterial genomic DNA (Megan, China). Using agarose gel electrophoresis and Qubit, the quality and quantity of the DNA was detected (Thermo Fisher Scientific, USA). After premixing using NEBnext-Ultra-II-Q5-Master-Mix, the V3-V4 region of the 16S rRNA gene was amplified (NEB, USA).

### Illumina sequencing

Agilent 2200 Tape Station and Qubit® were used to assess the final library product (Life Technologies, USA). The first batch of samples was sequenced on the Miseq (Illumina, USA) platform at with pair-end 250 bp, whereas the second batch was sequenced on the NovaSeq (Illumina, USA) platform at with pair-end 250 bp.

### Data analysis

The mean with SD was showed for normally distributed parameters, and *p* values were calculated using a student’s t-test; for not normally distributed parameters, the median with IQR (P25, P75) was showed, and *p* values were calculated using the Mann-Whitney U test. Counts were used to represent categorical variables, and the Chi-square test was used to obtain the *p* value. Statistical significance was defined as a value of *p* < 0.05.

Using the qiime tools import program, raw data FASTQ files were imported into a format that could be used by the QIIME2 system. To obtain the feature table of amplicon sequence variant (ASV), demultiplexed sequences from each sample were quality filtered and trimmed, de-noised, merged, and then the chimeric sequences were identified and removed using the QIIME2 dada2 plugin [21]. To generate the taxonomy table, the QIIME2 feature-classifier plugin was used to align ASV sequences to a pre-trained GREENGENES 13_8 99% database (trimmed to the V3-V4 region bound by the 338F/806R primer pair) [22]. The core-diversity plugin in QIIME2 was used to calculate diversity metrics. Wilcoxon calculated feature-level alpha diversity indices, such as Chao1 and Shannon diversity index, to estimate microbial diversity within a single sample. To study the structural variation of microbial communities among samples, researchers used beta diversity distance measurements and Bray Curtis, which were then visualized using principal coordinate analysis (PCoA) [23]. LEfSe was used to identify bacteria with varying abundances in different samples and groups [24] Clinical parameters and microbial species Spearman correlation coefficients were calculated and displayed as heat maps. The R program heatmap package is mostly used to generate the correlation heat map. PICRUSt was also used to predict the potential KEGG Ortholog functional profiles of microbial communities [25]. After getting the functional annotation, the Dunn test (R program dunn.test package) was performed to see if there were any significant differences between groups in the microbial community prediction function. A statistically significant value of *p* < 0.05 was used. The online sketching website was used to implement all visualizations (https://www.bioincloud.tech).

## Results

### Clinical characteristics of participants

In terms of age (*p* = 0.051), menopausal status (*p* = 0.798), BMI (*p* = 0.771), WHR (*p* = 0.243), SBP (*p* = 0.701), DBP (*p* = 0.127), hypertension, or diabetes (all *p* > 0.05), there was no significant difference between the P and H groups, indicating that the influence of gut microbiota caused by age, nutritional status, hypertension, or diabetes could be excluded (Table 1) However, the P groups had higher FSH and LH values, whereas the E2 levels were lower (all *p* < 0.05).

**Table 1:** Baseline characteristics

Parameters	H *n* = 24	P *n* = 77	***p*** value
Age (years)	47.88 ± 3.04	49.55 ± 3.77	0.051
Menopausal state n(%)			
Premenopausal	18 (66.67%)	47 (61.04%)	0.798
Postmenopausal	8 (33.33%)	30 (38.96%)	
BMI (kg/m^2^)	22.55 ± 1.26	22.43 ± 2.36	0.771
WHR	0.82 ± 0.04	0.81 ± 0.05	0.243
SBP (mmHg)	114.42 ± 11.52	116.00 ± 16.85	0.701
DBP (mmHg)	74.42 ± 6.15	77.42 ± 11.37	0.127
Hypertension, n (%)	0 (0.00%)	3 (3.90%)	1.000
Diabetes, n (%)	0 (0.00%)	1 (1.30%)	1.000
FSH (IU/L)	7.91 (4.80, 24.54)	68.46 (54.93, 85.64)	<0.05*
LH (IU/L)	5.45 (4.42, 15.84)	37.33 (28.33, 52.27)	<0.05*
E2 (pmol/L)	216.5 (109.42, 381.90)	51.65 (32.23, 77.47)	<0.05*

For normally distributed parameters, the mean was showed ±SD, and *p* values were calculated using a student’s t-test and for not normally distributed parameters, the median with IQR (P25，P75) was showed, and the *p* value was calculated using Mann-Whitney U test. Categorical variables are represented by counts, and *p* value was calculated using Chi-square test

*H* healthy controls, *P* Patients with MPS, *BMI* body mass index, *WHR* waist hip ratio, *SBP* systolic blood pressure, *DBP* diastolic blood pressure, *FSH* follicle stimulating hormone, *LH* luteinizing hormone, *E2* estradiol

*p < 0.05

### Analysis of gut microbiota diversity

**Fig. 1:**
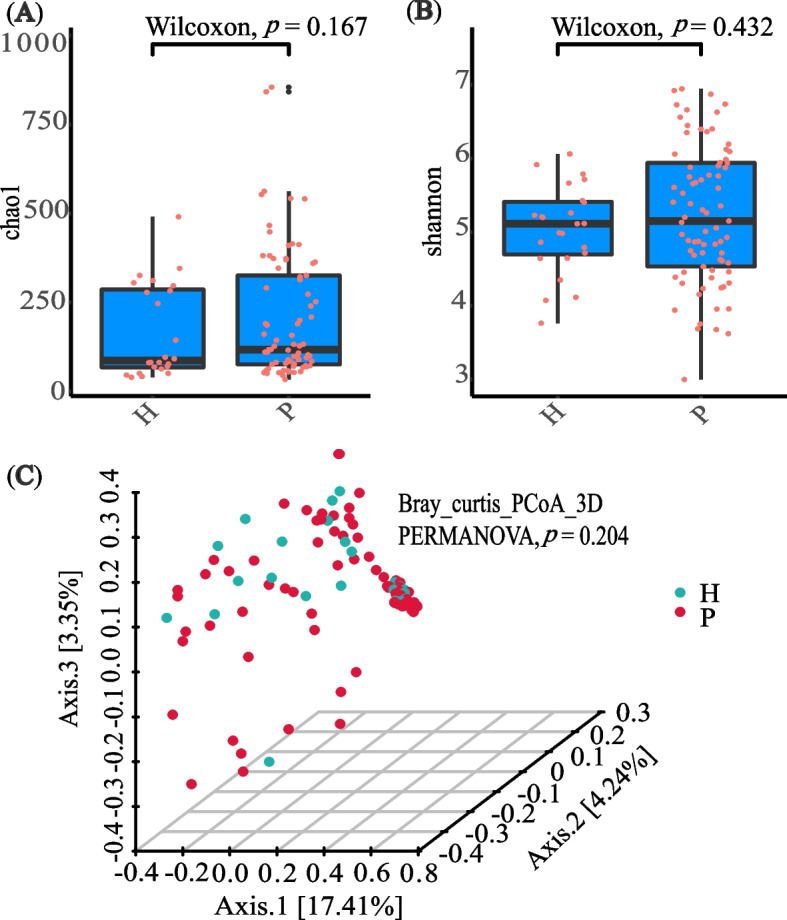
Gut microbiota diversity of patients with MPS (P group) and menopausal healthy women (H group) at menopause. **A** Alpha diversity was measured by both Chao1 and Shannon index for comparisons between P (*n* = 77) and H groups (*n* = 24). **B** Principal coordinate analysis (PCoA) with bray-curtis showed that P group (*n* = 77) had no significant difference from H group (*n* = 24)

In an analysis of gut microbial diversity, no significant differences were found between P (*n* = 77) and H group (*n* = 24). The sequencing depths were examined by plotting the rarefaction curve of richness (ggplot2) (Additional file 2). Each group’s curve was found to be near saturation, indicating that the sequencing depth was adequate. The abundance and diversity of microbial communities, as measured by Chao1 and Shannon index, were reflected in Alpha diversity. Between the two groups, there was no significant difference in Chao1 (Wilcoxon, *p* = 0.167) or Shannon index (Wilcoxon, *p* = 0.432) (Fig. 1A-B). Bray curtis calculated beta diversity, which is an indicator of microbial community composition. P group had no significant difference from H group (PERMANOVA, *p* = 0.204) (Fig. 1C), according to principal coordinate analysis (PCoA).

### Gut microbiota composition and difference analysis

**Fig. 2:**
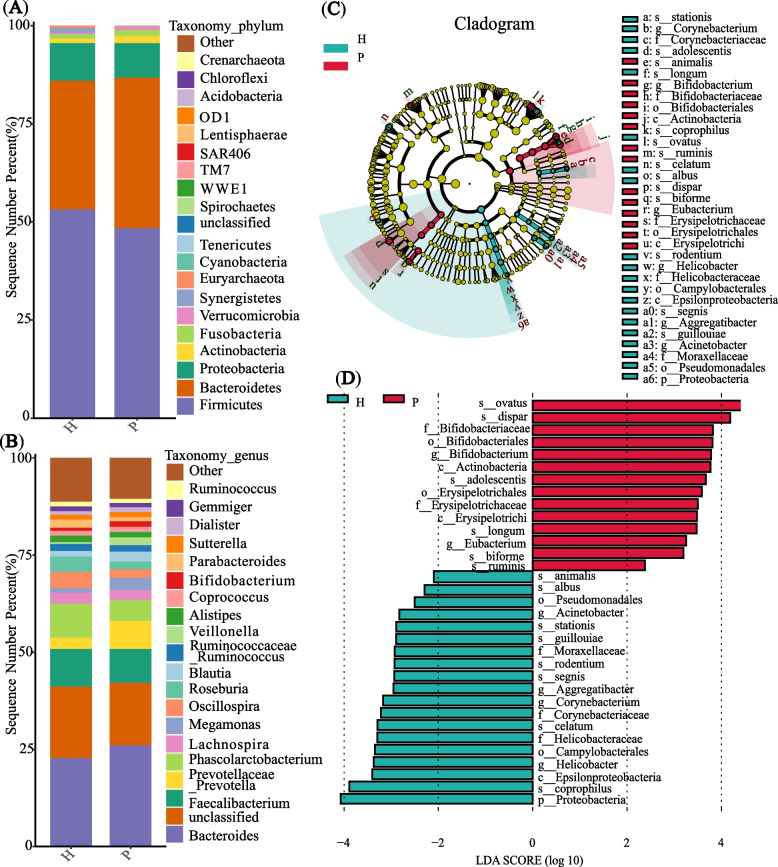
Composition and difference of the gut microbiota in P and H group. **A** Average relative abundance of microbiota at the phylum level and (**B**) at the genus level in P (*n* = 77) and H group (*n* = 24); sequences without annotations were classified as unclassified; those bacteria whose relative abundance was ranking > 20 were classified as others. **C** The taxonomic cladogram was generated based on the LEfSe and LDA scores. Bacterial taxa enriched in P group (red dots) and H group (green dots). **D** Taxa enriched in P group were indicated with a positive LDA score (red) and negative LDA score (green), respectively. Only taxa with an LDA significance threshold > 2.0 were showed in the figure; p, phylum; c, class; o, order; f, family; g, genus; s, species

In the P (*n* = 77) and H (*n* = 24) groups, a total of 43 phyla, 281 genera, and 163 species were identified (Additional file 3). At the phylum and genus level, the top 20 average relative abundance of microbiota were showed (Fig. 2A-B). Using LEfSe analysis, we compared the gut microbiota compositions in two groups. LEfSe analysis detected 14 species with varying abundances: In the P group, *Bifidobacterium adolescentis*, *Bifidobacterium longum*, *Bacteroides ovatus*, *Lactobacillus ruminis*, *Veillonella dispar*, and *Eubacterium biforme* showed greater enrichment, whereas in H group, *Corynebacterium stationis*, *Bifidobacterium animalis*, *Bacteroides coprophilus*, *Clostridium celatum*, *Ruminococcus albus*, *Helicobacter rodentium*, *Aggregatibacter segnis*, *Acinetobacter guillouiae* were more abundant (LDA significance threshold > 2.0; Fig. 2C-D). In conclusion, we identified different species in two groups, indicating a significant different composition of the gut microbiota between the P and H group.

### Associations between clinical parameters and gut microbiome

**Fig. 3:**
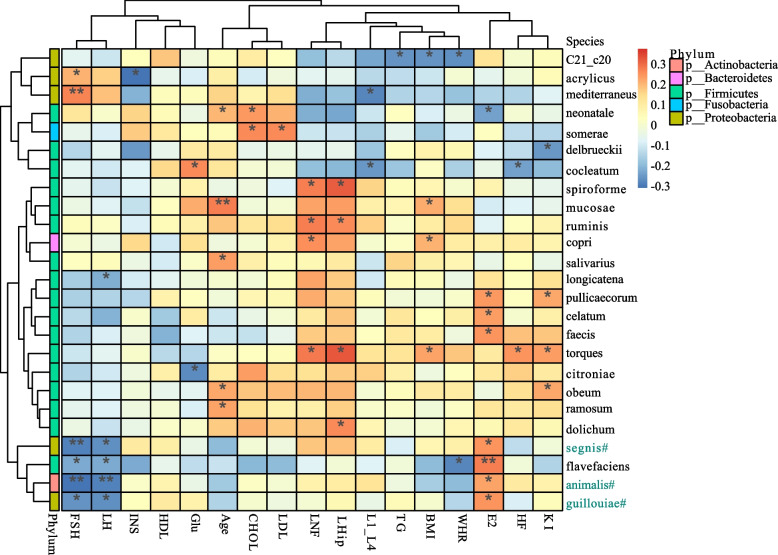
Correlation matrix for clinical parameters and species. Yellow and red signified a positive correlation, while blue signified a negative correlation. * 0.01 ≤ *p*<0.05，** 0.001 ≤ *p*<0.01. #Significantly different species between P and H groups, green signified genus enriching in H group. L1-L4, BMD of the 1st to 4th lumbar vertebrae; LNF, BMD of left neck of femur; LHip, BMD of the left hip; HF, Hot flash symptom score (a component of the domestic modified KI Score)

We explored the correlation between clinical parameters and species abundance using Spearman’s correlation analysis (Fig. 3). It was discovered that E2 had a positive correlation with *Aggregatibacter segnis*, *Bifidobacterium animalis* and *Acinetobacter guillouiae*, while FSH and LH had a negative correlation with these three species. E2 had the strongest positive correlation with *Acinetobacter guillouiae* (*r* = 0.253, *p* = 0.018), followed by *Aggregatibacter segnis* and *Bifidobacterium animalis*. FSH  (*r* = − 0.302, *p* = 0.004) and LH (*r* = − 0.276, *p* = 0.009) had the strongest negative correlations with *Bifidobacterium animalis*, followed by *Aggregatibacter segnis* and *Acinetobacter guillouiae*. Meanwhile, *Aggregatibacter segnis*, *Bifidobacterium animalis*, and *Acinetobacter guillouiae* were found enriching in the H group (LDA significance threshold > 2.0; Fig. 2C-D). The domestic modified KI scores was found to be positively correlated with *Ruminococcus torques*, *Blautia obeum* and *Butyricicoccus pullicaecorum*, while negatively correlated with *Lactobacillus delbrueckii*. The hot flash (HF) symptom scores was found to be positively related to *Ruminococcus torques*, while negatively related to *Clostridium cocleatum*. BMI, WHR, Glu, INS, CHOL, LDL, TG, and BMD were also correlated with species abundance. As a result, the clinical parameters were found to be correlated with the composition of the gut microbiome.

### Functional alternation of gut microbiota

Gut microbial functions were predicted by PICRUST in our study, of which the profiles revealed significant alterations in two groups. We constructed functional profiles for each sample using microbial KEGG Ortholog pathways. 8 KEGG level2 pathways (*p* < 0.05; Table 2) and 50 KEGG level3 pathways (*p* < 0.05; Additional file 4) enriched in the P group, were found to be significantly higher than in H group. Circulatory system, carbohydrate metabolism, digestive system, cell motility, signal transduction, folding, sorting and degradation, infectious disease, and biosynthesis of other secondary metabolites had more abundance in the P group than in the H group. Importantly, metabolic pathways related to cardiovascular disease and carbohydrate metabolism were enriched in the P group (*p* < 0.05; Table 3), indicating that the incidence of cardiovascular disease, obesity, diabetes and other related diseases may increase after women enter menopause.

**Table 2:** KEGG level2 differential pathways between P and H group

Feature	Expression	*Z*	*p* value adjusted
Circulatory system	P>H	−2.138	0.016*
Carbohydrate metabolism	P>H	−1.995	0.023*
Digestive system	P>H	−2.130	0.017*
Cell motility	P>H	2.019	0.022*
Signal transduction	P>H	1.939	0.026*
Folding, sorting and degradation	P>H	2.290	0.011*
Infectious disease: viral	P>H	2.098	0.018*
Biosynthesis of other secondary metabolites	P>H	−1.723	0.042*

The Dunn test was used to analyze whether the microbial community prediction function was significantly different between groups. Z, tests statistic of Dunn test

*p < 0.05

**Table 3:** KEGG level3 differential pathways between P and H group

Feature	Expression	*Z*	*p* value adjusted
Adrenergic signaling in cardiomyocytes	P>H	− 2.023	0.022*
Cardiac muscle contraction	P>H	− 1.947	0.026*
Dilated cardiomyopathy (DCM)	P>H	−1.727	0.042*
Arrhythmogenic right ventricular cardiomyopathy	P>H	−2.079	0.019*
Hypertrophic cardiomyopathy (HCM)	P>H	−1.823	0.034*
Calcium signaling pathway	P>H	−2.023	0.022*
cAMP signaling pathway	P>H	−2.047	0.02*
Carbohydrate digestion and absorption	P>H	−2.011	0.022*
Fructose and mannose metabolism	P>H	−2.162	0.015*
Galactose metabolism	P>H	−2.138	0.016*
Pentose and glucuronate interconversions	P>H	−2.513	0.006*
Insulin secretion	P>H	−2.023	0.022*
NOD-like receptor signaling pathway	P>H	1.939	0.026*

The Dunn test was used to analyze whether the microbial community prediction function was significantly different between groups. Z, tests statistic of Dunn test

*p < 0.05

## Discussion

The findings showed that the gut microbiome composition was altered in MPS patients: the abundance of 14 species differed significantly between MPS patients and menopausal healthy women. We found underlying and intriguing relationships between gut microbe composition and function and MPS. It was discovered that E2 had a positive correlation with *Aggregatibacter segnis*, *Bifidobacterium animalis*, and Acinetobacter guillouiae (these three species were enriched in menopausal healthy women), while FSH and LH had a positive correlation with them. The domestic modified KI score, HF symptom score, BMI, WHR, Glu, INS, CHOL, LDL, TG and BMD were also correlated with species abundance. Functionally, the MPS was enriched in metabolic pathways related to cardiovascular disease and carbohydrate metabolism, implying that the incidence of cardiovascular disease, obesity, diabetes, and other related diseases may rise at menopause.

Ovarian function declines as women enter menopause, and the ovaries produce less estrogen, so the level of estradiol in the blood drops and the level of FSH rises. Estrogens and other female hormones play a key role in regulating the gut microbiome’s composition [26, 27]. Premenopausal women’s estrogen levels are usually higher than postmenopausal women’s. A previous study found that pre-menopausal and post-menopausal women had significantly different estrogen levels and hormone secretion, which could be linked to changes in the gut microbiome [28, 29]. Our study showed that patients with MPS had lower levels of estradiol, higher levels of FSH and LH, and a significantly different gut microbiota composition than menopausal healthy women. These changes suggested that estrogen levels, FSH, and LH may have an effect on gut microbiota composition.

Estrogen has been showed to influence the gut microbiome, and gut microbiome also had a significant impact on estrogen levels [30, 31]. We found that three bacteria species (*Aggregatibacter segnis*, *Bifidobacterium animalis*, and *Acinetobacter guillouiae*) were enriched in menopausal healthy women, and E2 had a positive correlation with them, while FSH and LH had a negative correlation with them. *Bifidobacterium_animalis* is considered a probiotic that can improve abdominal obesity [32], inflammation, oxidative stress, blood lipids, blood sugar and vascular endothelial function in patients with metabolic syndrome [33]. *Aggregatibacter segnis* and *Acinetobacter guillouiae* are primarily related with inflammation and infections [34, 35]. Changes in sex hormone levels may be caused by a decrease of *Bifidobacterium animalis* in MPS. Previous studies found that most gut microbiome showed an increase of β-glucuronidase enzyme activity [36]. β-glucuronidase could inactivate estrogen by blocking its binding to glucuronic acid, thus increasing the amount of estrogen in the body [37]. The gut microbiota with a positive correlation with the E2 may have an increase of β-glucuronidase enzyme activity to increase the amount of estrogen in the body.

The decline in estrogen result in MPS with vasomotor symptoms (hot flash), sleep disturbances and insomnia, adverse mood, vulvovaginal atrophy, sexual dysfunction, et al. [38]. This is in line with menopausal hormone therapy’s rapid resolution of MPS. A new vaginal cream containing visnadine (0.30%), prenylflavonoids (0.10%) and bovine colostrum (1%) was able to ameliorate both vaginal health and sexual quality [39]. Because prenylflavonoids exert similar effect as estrogen [40], visnadine ameliorate female sexual arousal disorder [41] and bovine colostrum relieve vaginal dryness [42]. Menopause symptoms not only have a detrimental impact on one’s quality of life, but may also link patients to cardiovascular disease, diabetes, osteoporosis, and breast cancer [5–8]. The decline in estrogen is also a serious threat to physiological activities and correlates with diseases such as type 2 diabetes [43], obesity [44], cardiovascular disease [45] and osteoporosis [46].

Changes in estrogen and gut microbiota in patients with MPS are consistent with functional predictions. Estrogen plays a leading role in the causes of female obesity [47]. Estrogen binding to its receptor can regulate glucose and lipid metabolism in a variety of ways. Disturbances in these metabolic pathways would contribute to the development of metabolic syndrome in post-menopausal women, as well as an increased risk of cardiovascular disease [48, 49]. In estrogen-deficient rats, MPS can be alleviated by maintaining gut microbial diversity [50]. Current studies have suggested the potentially strong association between gut microbiota, bone remodeling and bone metabolic diseases [51]. Gut microbiota disorders may cause increased gut permeability and trigger activation of key inflammatory pathways for inducing bone loss in sex steroid-deficient mice [52]. Probiotics have shown a positive effect on the management of healthy bone [53]. A previous study showed that a high-fat/carbohydrate diet programmed the gut microbiota to be predominated by *Firmicutes (Clostridium)*, *Prevotella* and *Methanobrevibacter* but deficient in beneficial genera/species such as *Bacteroides*, *Bifidobacterium*, *Lactobacillus* and *Akkermansia* [54]. *Bifidobacterium animalis* abundance decreased in MPS, and metabolic pathways related to cardiovascular disease and carbohydrate metabolism were enriched in the MPS, according to our findings. Altering the gut microbiota in MPS patients may have therapeutic effects as well as reducing the risk of long-term chronic disease, according to our speculation.

## Conclusion

In conclusion, we discovered taxonomic signatures associated with MPS in the gut microbes and predicted their function. We propose a hypothesis about how the gut microbiome affects menopausal women based on our findings. In MPS, our findings revealed a dysbiosis of the gut microbiome. In menopausal women, *Bifidobacterium animalis* is likely to be a beneficial gut microbiota. The gut microbiota may produce β-glucuronidase, which increases estrogen levels in the body. MPS was found to be particularly rich in metabolic pathways related to cardiovascular disease and carbohydrate metabolism, implying that the incidence of cardiovascular disease, obesity, diabetes, and other related diseases rises as women approach menopause. In patients with MPS, altering the gut microbiota could have therapeutic effects as well as reducing the risk of long-term chronic disease. Nevertheless, several limitations of the study should be taken into account: first of all, the sample size is limited; second, more research is needed to determine whether probiotics and fecal transplantation are preferentially used to prevent potential risks in postmenopausal women; third, future multiomic studies with a larger longitudinal cohort, as well as animal model experiments, will be required to verify our findings and gain a better understanding towards the underlying mechanisms of gut microbiota in MPS. The findings of the gut microbiome study provide not only new insights into disease mechanisms, but also novel therapies to help women feel better after menopause.

## Supplementary Information

**Additional file 1.** Subject survey. Including general information of participants and the domestic modified Kupperman Index score.

**Additional file 2.** The rarefaction curve of richness in different groups. (A-B) The curve in each group is nearly smooth with a sufficient amount of sequencing data and few new undetected genes.

**Additional file 3. **Gut microbiota composition of phyla, genera, and species. There are 43 phyla, 281 genera, and 163 species were identified in the P (*n* = 77) and H (*n* = 24) groups.

**Additional file 4.** KEGG level3 pathways of functional prediction. 50 KEGG level3 pathways enriched in the P group were found to be significantly higher than in H group.

## Data Availability

The datasets generated for this study can be found in NCBI with accession code SRA: PRJNA858179 (https://www.ncbi.nlm.nih.gov/sra/?term=PRJNA858179).

## Abbreviations

MPS: Menopausal syndrome
16S rRNA: 16S ribosomal RNA
P group: Patients with menopausal syndrome
H group: Healthy women at menopause
BMI: Body mass index
WC: Waist circumstance
WHR: Waist hip ratio
SBP: Systolic blood pressure
DBP: Diastolic blood pressure
FSH: Follicle stimulating hormone
LH: Luteinizing hormone
E2: Estradiol
BMD: Bone mineral density
CHOL: Total cholesterol
HDL: High-density lipoprotein cholesterol
LDL: Low-density lipoprotein cholesterol
TG: Triglycerides
Glu: Fasting blood glucose
INS: Fasting insulin
L1-L4: BMD of the 1st to 4th lumbar vertebrae
LNF: BMD of the left neck of femur
LH ip: BMD of the left hip
KI: Kupperman index
HF: Hot flash symptom score (a component of the domestic modified KI Score)
