# Supplementary material for: The relationship between menopausal syndrome and gut microbes
Source: BMC Womens Health. 2022 Nov 8;22:437. doi: 10.1186/s12905-022-02029-w (PMC9644609; doi:10.1186/s12905-022-02029-w)
Supplement: Supplementary file 1 — Additional file 1. Subject survey. Including general information of participants and the domestic modified Kupperman Index score. [file 12905_2022_2029_MOESM1_ESM.docx]

Subject survey

1.General information

| Gender: □Female □Male | Age: |
| --- | --- |
| Marriage: | Height: cm |
| Weight: kg | BMI: kg/m^2^ |
| Blood pressure: / mmHg | Heart rate: Times/min |
| Waistline: cm | Abdominal circumference: cm |
| Hipline: cm | Waist hip ratio: |
| Occupation: | Education: |
| Smoke：□Yes □No | Drink wine：□Yes □No |

2.History of Present Illness

| Chief complaint: | Degree of illness: □Mild □Moderate □Severe |
| --- | --- |
| Treatment history: □Yes □No  Medication treatment:   1. Product name: Dose: Administration time: Deactivated: | |
| Allergy history:□Yes □No (If yes, please specify as follows: ) | |

3.Past history

| Tuberculosis:□Yes □No | Hepatitis:□Yes □No |
| --- | --- |
| Hypertension:□Yes □No | Coronary heart disease:□Yes □No |
| Apoplexy:□Yes □No | Cerebral hemorrhage:□Yes □No |
| Diabetes:□Yes □No | Other:□Yes（Please specify: ）□No |
| If there is any medication, please indicate:   1. Product name: Dose: Administration time: Deactivated: | |

4.Childbearing history and Menstrual history

| Pregnancy times: | Natural labour times: |
| --- | --- |
| Cesarean section times: | Artificial abortion times: |
| Menstrual cycle | Last menstruation: |
| Previous menstruation: | Amenorrhea:□Yes □No |

| **Domestic modified Kupperman Index score** | | | | | | |
| --- | --- | --- | --- | --- | --- | --- |
|  |  |  |  |  |  |  |
| Symptom | Basic score | Score degree | | | | Symptom score |
|  |  | 0 point  symptomless | 1 point Occasional symptoms | 2 points Symptoms persist | 3 points  Impact on life |  |
| 1.Hot flashes and sweating | 4 | symptomless | ＜ 3 times/day | 3-9 times/day | ≥10times/day |  |
| 2.Insomnia | 2 | symptomless | Occasional | Often, sleeping pills are effective | Affect work and life |  |
| 3.Fatigue | 1 | symptomless | Occasional | It's difficult to get to the fourth floor | Limited daily activities |  |
| 4.Emotional sensitivity | 2 | symptomless | Occasional | Frequent and restrained | Often, unable to restrain |  |
| 5.Headache | 1 | symptomless | Occasional | Often, tolerable | Need to take medicine |  |
| 6.Vertigo | 1 | symptomless | Occasional | Often, without affecting life | Affect daily life |  |
| 7.Palpitation | 1 | symptomless | Occasional | Often, without affecting life | Need treatment |  |
| 8.Urinary system infection | 2 | symptomless | Occasional | >3 times/year, self healing | >3 times/month, medication required |  |
| 9.Sexual life status | 2 | symptomless | Decreased sexual desire | Sexual difficulties | Loss of sexual desire |  |
| 10.Musculoskeletal pain | 1 | symptomless | Occasional | Frequently, without affecting functions | Dysfunction |  |
| 11.Formication | 1 | symptomless | Occasional | Often, tolerable | Need treatment |  |
| 12.Sensory disturbance | 2 | symptomless | Weather related | It usually feels cold, hot, painful and numb | Loss of feeling of cold, heat and pain |  |
| Total score |  |  |  |  |  |  |
| Degree evaluation | Normal Mild Moderate Severe | | | | | |
|  |  |  |  |  |  |  |
|  | Symptom score=Basic score × Score degree. The total score is the sum of all symptom scores. Degree of illness: Mild: 15-20 points; Moderate: 21-35 points; Severe:>35 points. | | | | | |
