# Supplementary figures and images for: The relationship between menopausal syndrome and gut microbes
Source: BMC Womens Health. 2022 Nov 8;22:437. doi: 10.1186/s12905-022-02029-w (PMC9644609; doi:10.1186/s12905-022-02029-w)

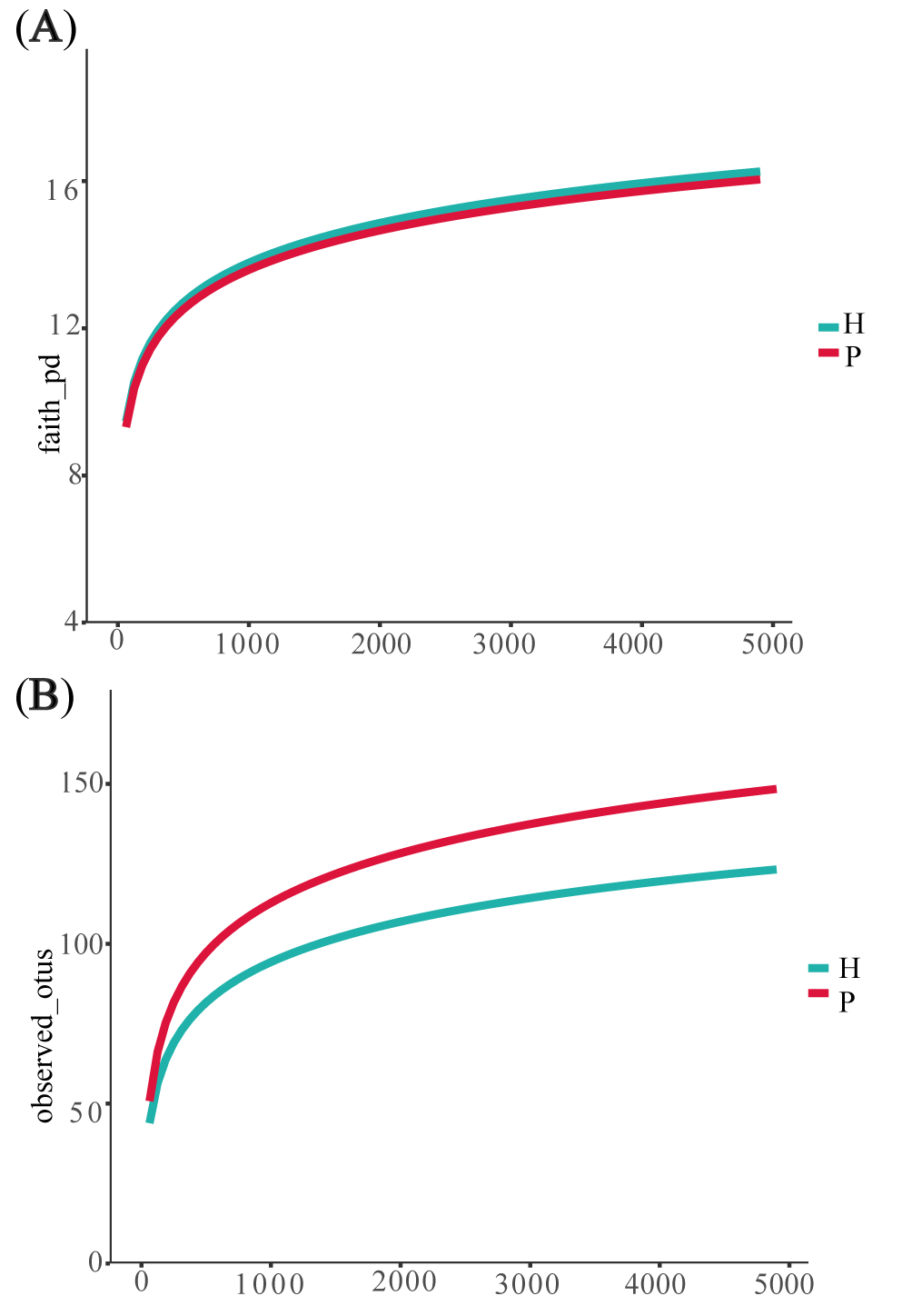

Supplement: Supplementary file 2 — Additional file 2. The rarefaction curve of richness in different groups. (A-B) The curve in each group is nearly smooth with a sufficient amount of sequencing data and few new undetected genes. [file 12905_2022_2029_MOESM2_ESM.tif]
